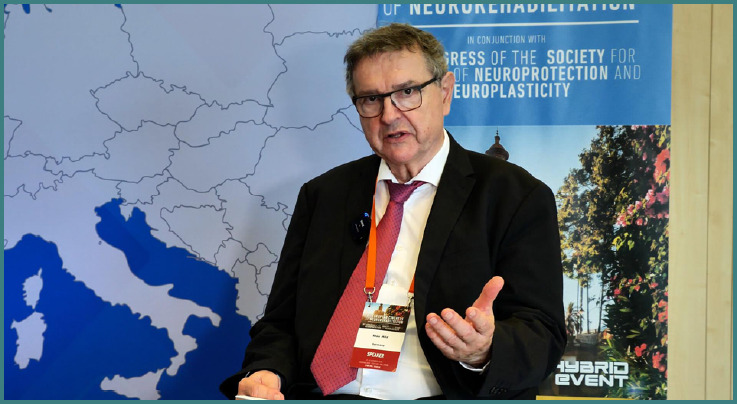# Interview with Prof. Max Hilz - 8^th^ European Congress on Neurorehabilitation in conjunction with the 20^th^ Congress of the Society for the Study of Neuroprotection and Neuroplasticity

**DOI:** 10.25122/jml-2026-1003

**Published:** 2026-01

**Authors:** Stefana-Andrada Dobran, Alexandra Gherman

**Affiliations:** 1 RoNeuro Institute for Neurological Research and Diagnostic, Cluj-Napoca, Romania; 2 Sociology Department, Babes-Bolyai University, Cluj-Napoca, Romania

**Interviewee: Professor Max Hilz**

**Interviewer: Ms. Stefana-Andrada Dobran**

Professor Max J. Hilz is a leading global authority in autonomic neurology, with a distinguished clinical and academic career in the United States, the United Kingdom, and Germany. His work has been defined by pioneering research into disorders of the autonomic nervous system, as well as sustained leadership in shaping the field internationally. He has chaired major autonomic and neurological bodies from major organizations, such as the World Federation of Neurology, the European Academy of Neurology, and the American Academy of Neurology, and has served as President of both the German Autonomic Society and the European Federation of Autonomic Societies. Professor Hilz has played a key role in establishing international subspecialty standards, including contributing to the development of the Autonomic Disorders subspecialty examination of the UCNS. He has authored hundreds of scientific publications and co-authored influential clinical guidelines on conditions such as syncope, orthostatic hypotension, and diabetic neuropathy. His work continues to shape both contemporary clinical practice and the next generation of autonomic neurologists.

**S.D.: Hello, Professor Max Hilz and welcome to the 8^th^ European Congress on Neurorehabilitation in conjunction with the 20^th^ Congress of the Society for the Study of Neuroprotection and Neuroplasticity. This European Congress of Neurorehabilitation brings together the scientific and clinical communities. What do you believe is the unique role that it plays in bridging the gap between research and daily patient care in neurorehabilitation?**

**
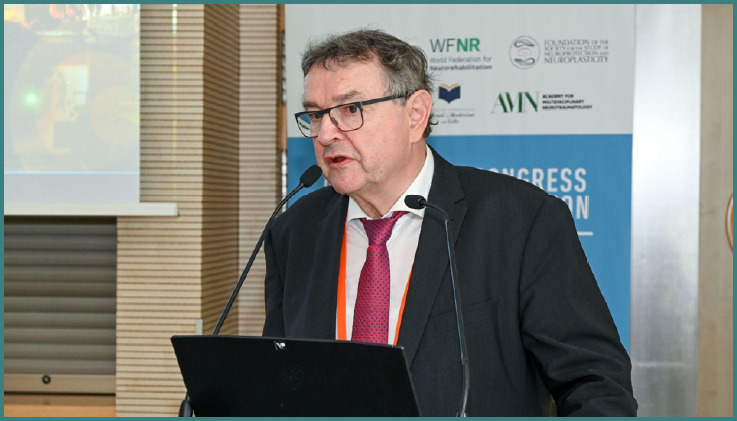
**

M.H.: First of all, thank you for having me. I think it is extremely important to have this conference because it builds a link between the many different subspecialties that exist in neurology and in neurorehabilitation itself. There are so many aspects like genetics, immunology, or my field, the autonomic nervous system, specialties and subspecialties which all play an important role in rehabilitation processes.

This conference, according to my perception, tries to address all these different important aspects, including novel aspects of neurorehabilitation, aspects some of which I, as a clinical neurologist, was not even aware of; for example, augmented reality that was the focus of one of the presentations we heard this morning, a technique that opens completely new doors for patients with movement disorders or with deficits after a stroke.

These are very new concepts, or at least new for me, that really advance the field. This includes music therapy, which is a field where we have also done some work on the autonomic nervous system effects of music therapy, but not on the effects in neurorehabilitation, which are very promising. There is a whole array of aspects that are covered in this conference and that's what makes the conference so interesting and links science to direct patient needs.

This link is, of course, of utmost importance because all the science would be *l'art pour l'art* unless the patients benefit from this work.

**S.D.: Considering your medical specialty, are there future developments that you envision for the complex multidisciplinary field of neurorehabilitation?**

M.H.: The autonomic nervous system interacts with all bodily functions, but unfortunately, not much autonomic research has so far been implemented in neurorehabilitation. This is because too many of our colleagues assume that the autonomic field is very complicated and needs expensive equipment. Therefore, it is very important to familiarize colleagues with the basic concepts of clinical autonomic evaluation and research and to introduce technologies that are not that difficult but still give us a prominent insight into the status of autonomic nervous system function—or in the course of a disease, autonomic dysfunction that requires further autonomic diagnosis and follow-up steps. This shows whether the status progresses and deteriorates or if there is an improvement.

For example, after stroke, we did studies where we saw—immediately after the stroke onset, within the first 24 hours—significant dysfunction of cardiovascular autonomic regulation. This correlates with the stroke severity and also with the compromised function of specific brain regions. Then, with recovery and rehabilitation, these parameters can improve. If they do not improve, this could indicate a high risk of continuous further secondary strokes, which, of course, deteriorates the patient's prognosis. So, these parameters should be implemented in rehabilitation medicine, and there is so much more that can be done.

Just think about the significant prevalence of bladder dysfunction in patients who need neurorehabilitation, be it due to multiple sclerosis, stroke, radiculopathy, etc. This dysfunction needs to be evaluated by autonomic examination. The examination is straightforward and quite simple to learn. I hope that all these aspects can be implemented. I just mentioned a few of them, but there are far more that could be implemented in order to better diagnose the status of a patient and then work on the improvement.

**S.D.: From your perspective, what is a challenging future development in neurorehabilitation that the EFNR can help with?**

M.H.: Well, there are many very challenging tasks. One task is, perhaps—just to pick an example—the improvement of motor function after a stroke. One of my friends who is also at this conference, Professor Wayne Feng from Duke University, uses repetitive transcranial magnetic stimulation to enhance motor function recovery and many other functions. I think this is a promising field which can, in the future, add much more to the rehabilitation of post-stroke patients.

Another option is vagus nerve stimulation, which can be performed non-invasively. There are now initial studies showing that motor function after brachiofacial hemiparesis can be improved if you apply adequate transcutaneous vagus nerve stimulation. There are definitely many other aspects which are exciting and deserve further research and clinical application for the benefit of the patients.

**S.D.: Do you think that the EFNR can support research or maybe advocacy for certain topics? Or what do you think is the biggest role the Federation can play?**

M.H.: A highly important part of research support includes financing research projects, but I don't think that financing research should be the task of a clinical and research society like EFNR. In my view, the most stimulating part of such a congress and a society like EFNR is the interaction between researchers who, unless they meet at such a conference, often don't even know that there is another group that pursues a similar goal, that there is an overlap between ongoing projects. This could trigger a mutually fruitful cooperation, which could give rise to joint studies and enhanced protocols.


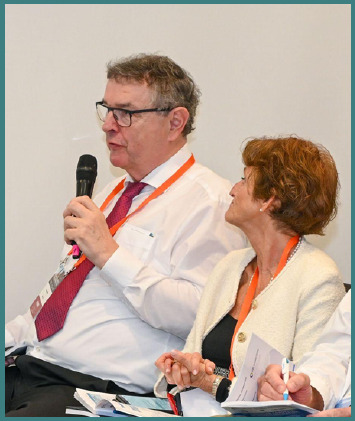


If I may refer to some of my own studies, I sometimes met people at conferences with whom I had a conversation during a coffee break or during dinner, and it turned out to be a stimulating exchange of ideas. These serendipitous conversations, in the end, triggered new research projects which somehow unraveled the Gordian knot. I don't think it makes sense to take a sword and cut the Gordian knot into two pieces. That's not a scientific solution. We have to come together at conferences like this one to unravel problems by exchanging thoughts and ideas.

Science is far more complicated than “solutions” that are considered heroic in history. We cannot just say, “Okay, we don't have a solution, we ignore the problem.” We have to untangle all these complicated and intricate problems. And that's what a society like EFNR will be able to advance: by exchanging ideas, by bringing people together, and by allowing them to share their ideas—and by exchanging them, creating new thoughts and gaining new insights.

**S.D.: Your pioneering work and your presentation today focus on the autonomic nervous system. How can a deeper understanding of autonomic regulation, for instance, after a stroke, transform our neurorehabilitation strategies and improve long-term patient outcomes?**

M.H.: First of all, so far—in my perception—rather little autonomic nervous system research and clinical evaluation is performed in neurorehabilitation patients. However, when you just look at the cardiovascular autonomic control, the relevance and predominance of autonomic dysregulation becomes obvious: cardiovascular autonomic function is controlled by the so-called central autonomic network that is located throughout the brain—with many centers in the brainstem, but also in many other brain areas like the insular cortex, frontal cortex, and anterior cingulate gyrus, and the multiple interconnections between all these autonomic centers.

When neurorehabilitation experts find an autonomic dysfunction in patients after a stroke, in patients who suffer from multiple sclerosis, in persons who had a brain injury, or patients who have epilepsy, they must know that a dysfunction in these central autonomic areas puts the patients at further risk. A lesion in one of the many central autonomic structures not only compromises normal organ function, such as normal cardiovascular function, bladder function, gastro-intestinal function; the patients might not be able to control their body temperature adequately, they might not be sweating or are profusely sweating. This is, of course, a major limitation of the afflicted patient’s quality of life.

If autonomic testing and evaluation can be implemented in rehabilitation—where you first assess the autonomic status and then follow up on it—it will enable us to improve the patient outcome and also the patient's quality of life. However, it is currently still one of the big challenges to train experts in neurorehabilitation in better understanding autonomic evaluation, autonomic nervous system function, and dysfunction.